# *Narratives of Discovery* as a catalyst for translational science education and training

**DOI:** 10.1017/cts.2025.60

**Published:** 2025-04-10

**Authors:** Leah G. Pope, Daichi Shimbo, Harold A. Pincus, Muredach P. Reilly, Rita Charon

**Affiliations:** 1 Irving Institute for Clinical and Translational Research, Columbia University Irving Medical Center, New York, NY, USA; 2 Department of Psychiatry, Vagelos College of Physicians and Surgeons, Columbia University Irving Medical Center, New York, NY, USA; 3 New York State Psychiatric Institute, New York, NY, USA; 4 Department of Medicine, Vagelos College of Physicians and Surgeons, Columbia University Irving Medical Center, New York, NY, USA; 5 Division of Cardiology, Department of Medicine, Columbia University Irving Medical Center, New York, NY, USA; 6 Department of Medical Humanities and Ethics, Vagelos College of Physicians and Surgeons, Columbia University Irving Medical Center, New York, NY, USA

**Keywords:** Translational science, education/training initiatives, creativity, innovation, workforce development

## Abstract

New education and training opportunities are critical for the development of a diverse and highly skilled translational science workforce. In this special communication, the authors consider how *Narratives of Discovery*, an initiative to interview leading scientists about the sources of their creativity, can serve as a novel translational science teaching tool. Reporting on a project to map translational science principles onto nine *Narratives of Discovery* conducted to date, the authors demonstrate how translational science principles are manifested in the career trajectories of these scientists and propose that the narratives can serve as a formative model for trainees. Findings from systematic coding of the *Narratives of Discovery* suggest that the narrative format is particularly well suited to highlight translational science principles not well-addressed by existing education opportunities, including what it means for scientists to be creative and innovative, use bold and rigorous approaches, and prioritize diversity, equity, inclusion, and accessibility. Offering excerpts from the published *Narratives of Discovery* and quotations from the scientists themselves, the authors aim to create space for continued conversation about how to best crystallize the concepts of translational science and advance translational science education and training initiatives.

## Introduction

Since 2021, the Irving Institute for Clinical and Translational Research (Irving Institute) at Columbia University Irving Medical Center has produced the *Narratives of Discovery* (*Narratives*) series with inspiration from the field of narrative medicine. Conceived as a project to highlight the creative dimensions of scientific knowledge, *Narratives* interviews today’s leading scientists about the sources of their ideas and hypotheses that move them toward discovery. *Narratives* has produced nine interviews to date with scientists from a range of fields including biomedical informatics, genetics and pediatrics, cancer immunology, physiology and pharmacology, social work and implementation science, systems biology, and translational medicine and therapeutics. The resulting narratives offer biopsies of individual scientific trajectories, capturing the ways in which these scientists “all rely on creative acts of imagination as well as on empirical replicable findings” [1].

The Irving Institute’s administrative core has engaged in strategic planning about *Narratives* to design next steps for the series and consider alignment with other strategic priorities. This process has led to a targeted focus on understanding whether and how the essays can be a vehicle for elucidating principles of translational science – the field of investigation that aims to distill the scientific and operational processes underlying each step of the translation process [2]. It has also stimulated reflection on identifying new opportunities for training translational scientists, including discussions of whether the narrative format itself can serve as a novel teaching tool. Here, we explain the narrative medicine technique and development of *Narratives* and report on the results of a project to map translational science principles onto the existing narratives. In so doing, we consider the specific utility of narratives for conveying principles of creativity, boldness, and equity, and reflect on how narrative writing might be harnessed in education and training initiatives. This manuscript will provide excerpts from the published *Narratives* and quotations from the scientists themselves about the creative processes that led to translational science breakthroughs. These verbatim communications by the scientist about how they incorporated one of the translational science principles may function as both aspirational models for scientists-in-training and as concrete instructions on how paradigm-shifting discovery occurs.

### Educating translational scientists

The relatively recent articulation of translational science as distinct from translational research has led to calls for building an education and training framework that can prepare the next generation of translational scientists [3,4]. In turn, the National Center for Advancing Translational Sciences (NCATS) has made substantial investments in formalizing the core knowledge that defines translational science. This includes the development of 14 clinical and translational research core competencies areas in 2011 [5,6], the codification of seven characteristics of translational scientists by the Translation Together consortium in 2019 [7], and the articulation of translational science principles by NCATS staff in 2020 [8]. The translational science principles are intentionally broad. They include: (1) prioritize initiatives that address unmet needs; (2) produce generalizable solutions for common and persistent challenges; (3) emphasize creativity and innovation; (4) leverage cross-disciplinary team science; (5) enhance the efficiency and speed of translational research; (6) utilize boundary-crossing partnerships; (7) use bold and rigorous research approaches; and, until early 2025, (8) prioritize diversity, equity, inclusion and accessibility (DEIA) [8,9].

Many institutions are now using these identified translational science principles to develop education and training opportunities. In 2020, NCATS developed an online case-study-based course in translational science for students across all education and career stages [10]. An evaluation indicated that it led to statistically significant increases in knowledge about translational science principles as well as attitudes about collaboration in translational research, suggesting that these principles can be taught using “real life” examples [10]. Multiple CTSA hubs now participate in a translational science case study working group to produce generalizable insights into the translational process by identifying key facilitators and barriers to translation and highlighting how to overcome challenges to achieve impact [11]. These efforts successfully expand the scope and content of education and training opportunities in translational science.

Vogel and colleagues conducted a scoping review and identified 29 articles describing current, previous, or planned translational science education and training opportunities (as well as 15 articles with recommendations for translational science education) [10]. Articles were systematically coded for education and training opportunities relevant to translational science principles. Though nearly all the articles (91%) included content related to more than one translational science principle, there was wide variation in how frequently each of the principles was represented. While three-quarters of articles reflected content on “utilizing boundary-crossing partnerships to advance translation” and “leveraging cross-disciplinary team science,” only 41% of articles referenced “emphasizing creativity and innovation” and only 7% of the articles referenced content about “engaging in evidence-informed risk taking” (since relabeled by NCATS as “using bold and rigorous approaches”). Further, the principle of creativity and innovation was more likely to be represented in articles that focused only on recommendations for translational science education (45%) rather than articles reporting on existing or planned education opportunities [10]. These findings led Vogel et al. to conclude that certain topics could benefit from further development in translational science education and training, including those focused on emphasizing creativity and innovation and engaging in bold and rigorous approaches.

### Narrative medicine and the development of Narratives of Discovery

*Narratives of Discovery* emerged from the discipline of Narrative Medicine, a humanities-based field that incorporates concepts and skills from literary, philosophical, and creative fields into bioscientific and clinical practices. Its principles and practices contribute to conceptualizing, articulating, and teaching issues of health and disease [12,13]. The Irving Institute hypothesized that well-written narratives of the work of influential investigators might display discovery in action as historical data and inspiration for scientists and trainees. They worked with general internist and literary scholar Rita Charon to develop the *Narratives* project and to select clinical and translational scientists at varying points in their careers at Columbia and elsewhere to tap for interviews. All scientists approached agreed to be interviewed.

After a detailed review of the subject’s investigations, Dr Charon conducted hour-long interviews once or twice with each scientist. She used a narrative inquiry approach instead of standardized questions to learn what attracted the scientists to their questions, what their igniting curiosities were, what led to their glimmers of discovery, and what were the stakes of the questions and their findings. In almost all interviews, subjects spoke about formative influences on their thinking from philosophy, history, literature, or the arts. Working from transcripts of the interviews, Charon assembled an account of the scientific questions and the scientist’s unique approach to trying to answer them, focusing always on the creative processes that led to new knowledge.

The essays were posted on the Irving Institute website and broadcast to institutional faculty and to NCATS nationally. Each article has had an average of 515 unique views in its first month of being posted (ranking second or third in overall traffic to the Columbia CTSA website) and the number of views has continued to grow, showing continued interest. Additionally, some *Narratives* were assigned as reading in medical school courses and described and quoted in publications [1].

We inaugurated a pilot of the educational use of the *Narratives* at the Columbia Vagelos College of Physicians & Surgeons MD/MS degree in Medical Science. Since 2022, Dr Charon and a *New York Times* science journalist offer an interviewing/writing course for 8-10 MD/MS students per year seeking to delve deeply into a particular area of their scientific interest. We train these students to interview an author of a seminal paper in the field and to write a narrative account of what they have learned. One or more of the *Narratives* essays are required reading for the course. These first-year medical students, most of them untrained as writers, receive 10 hours of classroom instruction, interview their chosen scientist, and shared their written draft of the interview with the class. They receive oral and written critiques from faculty and colleagues. Students craft complex, multi-dimensional narratives about their chosen scientists that incorporate technical material along with personal motives and biographical milestones of their subject.

Students in the course have reported that the writing enabled them to cohere and comprehend what they had learned from the interview. Not until they wrote their narrative could they appreciate how they had conceived of the story of the conversation. One student’s comments on the process, echoed by other students, sum up the fruits of the course: “I wanted to thank you all for the experience and taking the time to meet with us throughout the year. I’ve truly learned so much about writing, narrative structure, introspection, and story-telling through this program.” Some students in the course continue to develop narrative competence in their academic work, choosing challenging, non-reductive questions to investigate and obtaining further narrative training.

Although limited to medical students, this pilot supports the hypothesis that medical and scientific trainees without writing experience can learn in a short training course how to conceptualize and articulate their developing knowledge of complex science. Further, it suggests that this type of training can inspire excitement and further new kinds of engagement among cohorts of early career researchers.

### Methods: mapping translational science principles onto Narratives of Discovery

In order to elucidate areas of overlap between *Narratives* and translational science principles, a team at the Irving Institute chose to systematically code each interview in the series. The nine narratives written by Rita Charon that corresponded to nine interviews with leading scientists (see Table 1) were uploaded into Dedoose, a qualitative coding software. A codebook was created that included a code for each translational science principle followed by a definition as well as subcodes for example approaches as reflected on the NCATS website [14]. One researcher then coded each interview. Two additional codes were added during the coding process to reflect consistent themes in the interviews that are not captured explicitly by translational science principles: “mentorship” (to capture mentorship both received and given during the scientist’s career) and “early influences” (to capture formative experiences that scientists discussed as being impactful to their career trajectories). Results were presented to the Irving Institute CTSA administrative core team for feedback.

**Table 1.** *Narratives of Discovery* interviews

Name	Current title	Affiliation
Gordana Vunjak-Novakovic, PhD	University and Mikati Foundation Professor of Biomedical Engineering and Medical Sciences Laboratory for Stem Cells and Tissue Engineering	Columbia University Irving Medical Center
Andrea Califano, PhD	Clyde and Helen Wu Professor of Chemical Systems and Biology Director, Chan Zuckerberg Bio-Hub New York	Columbia University Irving Medical Center
Nabila El-Bassel, PhD	University and Willma and Albert Musher Professor of Social Work	Columbia University School of Social Work
Wendy Chung, MD, PhD	Mary Ellen Avery Professor of Pediatrics, Chair, Department of Pediatrics	Harvard Medical School Boston Children’s Hospital
George Hripcsak, MD, MS	Vivian Beaumont Allen Professor of Biomedical Informatics, Past Chair, Department of Biomedical Informatics	Columbia University Irving Medical Center
David Ho, MD	Professor of Microbiology & Immunology, Clyde ’56 and Helen Wu professor of Medicine, Director of Aaron Diamond AIDS Research Center	Columbia University Irving Medical Center
Henry Colecraft, PhD	John C. Dalton Professor of Physiology and Cellular Biophysics and Professor of Pharmacology	Columbia University Irving Medical Center
Garret FitzGerald, MD, FRS	Robert L. McNeil Jr. Professor in Translational Medicine & Therapeutics, Associate Dean for Translational Research, Director, Institute for Translational Medicine and Therapeutics, Senior Advisor to the EVP and Dean	University of Pennsylvania Perelman School of Medicine
Consuela Wilkins, MD, MSCI	Professor of Medicine, Senior Vice President and Associate Senior Dean for Health Equity and Inclusive Excellence, Co-Director of Clinical and Translational Research	Vanderbilt University

## Results

Coding of the interviews revealed evidence of all translational science principles. Similar to Vogel et al.’s [10] findings, examples of team science and boundary-cross partnerships were common in the *Narratives*. Yet, some of the most frequently encountered principles in our *Narratives* were those that have not been well-represented in educational and training initiatives: an emphasis on creativity and innovation, and the use of bold research approaches. We include the DEIA principle in our review even though its frequency was not tabulated by Vogel et al. because of its prominence in today’s translational science practices.

### Narratives of Discovery highlight translational science principles not well-addressed by current educational outreach

Here, we highlight evidence from *Narratives* that treat the under-represented principles, proposing that wider utilization of the *Narratives* methods would improve understanding and uptake of these under-taught aspects of translational science.

#### Emphasize creativity and innovation:

Andrea Califano, physicist and computational biologist, founded the Systems Biology Department at Columbia University and is now the Director of the multi-institutional Chan Zuckerberg Bio-Hub New York, a paradigm-transforming New York regional Biohub that will “bioengineer immune cells to scout, report, and repair damage to our cells before it leads to serious illnesses” [15].

Califano has helped to fundamentally shift biology toward ever more accurate and predictive maps of the inter-workings of the genetics, the epigenetics, the proteomics, and the outcomes of how cells work and even how to alter their workings to the benefit of our health. He studied the cancer cell’s capacity to maintain homeostatic control of its transcriptional products in the face of heterogeneous genetic mutations. “So we thought,” he said in his *Narratives* interview, “that in cancer there must be a piece of homeostatic control machinery responsible for integrating the effects of widely diverse mutational repertoires to implement a virtually identical, highly stable cell state.” Andrea’s imagination led him and his team to find Master Regulator Molecules in cancer cells, as they had predicted: “There’s nothing magical about what we do. It’s just that nobody had kind of thought of cancer in this way. . . . And this is not just in cancer. Because this is not only how cancer cells work but rather how virtually all human cells work.” Andrea *defamiliarizes* what he knows, in part achieved through his knowledge of philosophical antecedents, to consider novel aspects of the problem freed from prior assumptions.

Reflections on creativity abounded in many of these narratives. Biophysicist and pharmacologist at Columbia Henry Colecraft brings readers to the very edge of nano-scaled calcium ion channels in cardiomyocytes that control and synchronize the components of the heartbeat. “Sometimes it is helpful to make yourself small to, to, to imagine yourself at the scale of the channel.” Like the *Incredible Shrinking Man* science fiction movie, Colecraft brings himself imaginatively down to the scale of the very structure that he investigates. “I think it’s imagination. Imagination is seeing what’s not there. It’s a skill to see what’s not there… That is the rare quality that sets the scientists apart. I think imagination is key.”

#### Use bold and rigorous research methods:

Virologist and immunology researcher David Ho led the race to treat HIV/AIDS when, after a milestone-filled career in HIV research culminating in the development of HAART therapy, he and his team were faced with the events of January 2020. Dr Ho realized that his HIV Center’s virological research expertise conferred on it a scientific and even civic or ethical obligation to contribute to SARS-CoV2 research. In a *Narratives* interview, Dr Ho described how his team accomplished their work: “We not only could see but we could feel the impact of the pandemic. We lived it along with everybody else. My team largely moved into the [Columbia] dorm. . . . The hospital provided the meals to them. They worked anywhere between 12 and 16 hours a day. From February of 2020 through much of that year . . . they saw their families only occasionally.”

He knew he was leading his lab to break from ordinary practice: “I have taught my students and postdocs “Don’t accept dogma, dogma are not necessarily the truth . . . Learn to challenge dogma.”

#### Prioritize diversity, equity, inclusion, and accessibility (DEIA)

Prior to 2025, NCATS articulated the goal of enhancing DEIA in the translational workforce as a method of addressing persistent health inequities [16]. Consuelo Wilkins is an internist and co-director of Vanderbilt University’s CTSA and Senior VP and Senior Associate Dean for Health Equity and Inclusive Excellence. She explains that perspectives of community residents and members of the populations served by the researchers must be built in to the development of research questions, research design, and implementation strategies from the start of the research.

Her leadership is driven by her conviction that the problems of racial inequity in health care require attention to multiple factors at once. Social, cultural, historical, familial, psychological, economic, and clinical factors all have to be held in view at once were one to comprehend the phenomenon of racism in health care. “People need some mentoring, but that’s not all they need. They need a fair chance,…assessed [without bias],. . . [and] considered in light of the structural barriers [of]racism.”

## Discussion

*Narratives of Discovery* joins decades of scientific and arts-related investigations of the intersections between creativity and science [17,18]. Fundamental aspects of scientific discovery are found to mirror creativity in artistic and humanistic disciplines [18,19]. Scientific investigations of neural mechanisms of creativity demonstrate parallels between the brain activity of the creative work of scientists and visual artists [20]. Science educators internationally have championed the inclusion of arts and creativity for students from kindergarten to doctoral studies [21].

Literary scholars and philosophers have systematically articulated the methods they use to capture original creative ideas and to convey them to others [22,23]. Teachers of literature and writing have of late concentrated on the esthetic dimensions of what they teach their students, realizing that they pedagogically convey the esthetic powers of attention in addition to the structural or historical aspects of literary works [24]. Novelists and poets themselves dramatize and teach the powers of the imagination to display insights impossible to convey in the discourses of reason [25,27].

Rigorous training in creative sensibility and mastery of some creative skills may be valuable for the teachers of translational science trainees and for the trainees themselves. Both cohorts may need to expand their comprehension of the necessity for creative work within their science and their potential for performing creative work themselves. Accordingly, the *Narratives* project proposes pedagogic guidelines and instructional means to produce trainers and trainees who themselves are equipped with the wherewithal to fulfill the narrative, social, relational, and reflective dimensions necessary to live up to the standards and promise of translational science.

### Pedagogic theories and models

Conceptually, the interviews of *Narratives* follow the train of thought of the interviewed scientist. With the goal of exposing *what matters* to the investigator, the conversations uncover values, ideals, social vision, and scientific commitments of the interviewed scientist along with details of the science itself. The following pedagogic principles and practices guided the evolving process of producing the profiles:

1. *The Zone of Proximal Development (ZPD) and Creativity:* Learners learn best when situated at the boundary between what they know and what remains to be learned [28,29]. ZPD suggests that we aim our teaching and mentoring not only at senior investigators but also at junior faculty and trainees whose *modus* of inquiry is in formation.
2. *Principles of Mentorship:* Bioscience laboratories can either incubate or extinguish mentorship; intentional training can accelerate proficiency either as an effective mentor or an evolving mentee [30]. *Narratives* can contribute to the mentorship training process both by sharing the written portraits with trainees and also by coaching them in developing the skills of portraiture of their own.
3. *Primacy of Attention:* Neurocognitive sciences and poetic esthetics join in their endorsement of the primacy of attention and creative work’s capacity to expand attention [31,32]. Clinical medicine and scientific investigation are urgently aware of the wholesale decay of attention in the face of multi-media distractions and the requirements for speed set by corporate structures and funding agencies’ deadlines [33].
4. *Compositional rigor:* As a genre, *Narratives* require certain creative and cognitive skills of the author. A narrative pedagogy provides trainees and faculty with the tools of expository and creative writing to depict the scientist and to describe the significance of their work. Novice writers learn to adopt the tools of the essayist or novelist – structuring the plot, introducing the characters, using powerful metaphors – to comprehend and then communicate to the reader the importance of the work.

### Conclusion: methods for CTSA evolution of Narratives of Discovery

Unlike elementary- and high-school teachers of STEM disciplines, PIs and faculty mentors in the sciences do not obtain in-depth instruction in teaching their science [34]. They themselves may need tutoring in collaborative, creative, discovery-based science training. The *Narratives* project envisions a roadmap of pedagogically sound interventions for local and disseminated educational skills-building of both trainees and scientists.

We suggest a process through which individual or joined CTSAs might put this learning tool into action. Senior faculty and mentors could be introduced to the interviewing methods in writing *Narratives*, in either on-site training or at conferences. Junior faculty and trainees could use these reports to learn what matters to the scientists and what innovative and creative methods were used in the research.

Dividends emerge when junior faculty and mentees are trained to do the interviews themselves. As occurred in our pilot program, they will discover in the course of interviewing a scientist how the research findings emerge interwoven with life-long curiosities and social values and ideals. More powerfully, trainees will learn about both discovery and its unfolding throughout a scientist’s career when they become the *subjects* of an interview about their own work. They will articulate – and will therefore discover – what they themselves do in their work as scientists and what events in their own lives drew them to their particular questions.

Such processes may accomplish important goals. Individual trainees will gain clarity on their near and far goals. They may see aspects of their formation that may not have been consciously recognized. If done in lab groups, team cohesion among a cohort of trainees will likely be enhanced as they learn about one another’s lives in science. They may be proud of crossing the barrier between art and science and performing both throughout their translational lives.

On a broader level, the narrative approach can help to crystalize and communicate the concepts of translational science across the range of trainees, members of interdisciplinary research teams, and other stakeholder groups by publishing *Narratives* in clinical journals and basic science journals. In our determination to educate and inspire members of the lay public about the critical importance of clinical and translational science, we foresee that these stories and essays will attract public media editors to publish profiles of translational scientists in the lay press. A collaborative approach across the NIH, CTSAs, and NCATS may raise the prominence of translational sciences in the eyes of the public, ignite a recognition of translational science’s contributions to bioscientific discoveries, and facilitate creativity in science to a transformed landscape of health, wellbeing, and discovery.
